# Effect of Exercise Volume on Plantar Microcirculation and Tissue Hardness in People With Type 2 Diabetes

**DOI:** 10.3389/fbioe.2021.732628

**Published:** 2021-11-25

**Authors:** Weiyan Ren, Yijie Duan, Yih-Kuen Jan, Wenqiang Ye, Jianchao Li, Wei Liu, Hongmei Liu, Junchao Guo, Fang Pu, Yubo Fan

**Affiliations:** ^1^ Key Laboratory of Rehabilitation Technical Aids for Old-Age Disability, Key Laboratory of Human Motion Analysis and Rehabilitation Technology of the Ministry of Civil Affairs, National Research Center for Rehabilitation Technical Aids, Beijing, China; ^2^ Key Laboratory for Biomechanics and Mechanobiology of Chinese Education Ministry, Beijing Advanced Innovation Center for Biomedical Engineering, School of Biological Science and Medical Engineering, Beihang University, Beijing, China; ^3^ Rehabilitation Engineering Laboratory, Department of Kinesiology and Community Health, University of Illinois at Urbana-Champaign, Champaign, IL, United States; ^4^ School of Engineering Medicine, Beihang University, Beijing, China

**Keywords:** diabetic foot, weight-bearing exercise, plantar microcirculation, tissue hardness, exercise volume

## Abstract

**Objective:** Exercise has been reported to be beneficial for people with type 2 diabetes (T2DM), but exercise, especially weight-bearing exercise, may increase the risk of diabetic foot ulcers (DFUs). This study aimed to explore the associations between different volumes of weight-bearing physical activities and plantar microcirculation and tissue hardness in people with T2DM.

**Methods:** 130 elderly people with T2DM were enrolled for this cross-sectional study. They were classified into the high exercise volume group and the low exercise volume group based on their weekly energy expenditure (metabolic equivalents per week) in the past year. Weekly energy expenditure was calculated using the International Physical Activity Questionnaire and the Compendium of Physical Activities. The plantar oxygen saturation (SO_2_) and soft tissue hardness of each participant’s right foot were measured.

**Results:** A total of 80 participants completed the trial. The average exercise energy expenditure of the high exercise volume group and the low exercise volume group were significantly different (*p* < 0.05). The results showed that the SO_2_ of the high exercise volume group (67.25 ± 6.12%) was significantly higher than the low exercise volume group (63.75 ± 8.02%, *p* < 0.05). The plantar tissue hardness of the high exercise volume group was lower than the low exercise volume group in the big toe, midfoot and hindfoot regions (*p* < 0.05).

**Conclusion:** This study demonstrates that higher volumes of exercise are associated with better plantar microcirculation and lower plantar tissue hardness in people with T2DM. The findings of this study indicate that weight-bearing exercise may not increase risk of developing diabetic foot ulcers.

## Introduction

Diabetic foot ulcers (DFUs) are one of the most common and serious complications of diabetes mellitus (DM). A global survey on diabetes-related complications showed that one-third of people with diabetes suffered complications in the lower extremity (Zhang et al., 2020); and diabetes-related lower extremity amputations accounted for 30–65% of all amputations (Narres et al., 2017). DFUs can have a huge negative impact on the physical health and quality of life of people with diabetes.

Microvascular dysfunction (Greenman et al., 2005; Chao and Cheing, 2009), abnormal plantar stress (Jan et al., 2013b; Pu et al., 2018), increased plantar tissue hardness (Mithraratne et al., 2012; Jan et al., 2013a) and peripheral neuropathy (Bowering, 2001; Caselli et al., 2002) are major factors causing the development of DFUs. Research studies have shown that people with diabetes exhibit microvascular dysfunction, including a lower level of oxygen saturation of plantar tissue (Greenman et al., 2005; Chao and Cheing, 2009). Besides, the increased hardness of plantar tissue causes an increase in peak plantar pressure (Jan et al., 2013a; Teoh and Lee, 2020), which may gradually reduce the capacity to attenuate the ground impact in diabetic plantar tissue. Peripheral neuropathy can lead to a loss of protective sensation in people with diabetes, and also further aggravates microvascular dysfunction, as well as cause dry skin and musculoskeletal deformities (Armstrong et al., 2017). Although the relationships among oxygen saturation, plantar tissue hardness, neuropathy, and the occurrence of ulcerations are still unclear, these factors may play an important role in predicting and assessing the risk of DFUs.

Exercise is one of the most effective methods for managing the complications of diabetes, and has been shown to improve blood glucose levels, ankle brachial index, cardiopulmonary endurance, and muscle strength (Liao et al., 2019; Verboven et al., 2019). Moreover, weight-bearing exercise has been reported to improve tissue tolerance and significantly increase the achievable walking distance and step count of people with diabetic peripheral neuropathy (DPN) (Mueller and Maluf, 2002; Mueller et al., 2013; Kluding et al., 2017). Diloreto et al. also found that daily physical activity with an energy expenditure at 27 Mets·h/week (more than 10 Mets·h/week recommended by the American Diabetes Association) had a significant positive effect on the physical fitness of people with type 2 diabetes (Di Loreto et al., 2005; Association, 2020). However, the effects of exercise volumes of weight-bearing exercise on the risk of developing DFUs remain unclear (Liao et al., 2019). Exercise can improve endothelial function and blood circulation in the lower extremity (Mueller et al., 2013; Liao et al., 2019), which may be beneficial to improve microvascular function in people with type 2 diabetes (Mueller and Maluf, 2002; Kluding et al., 2017). On the other hand, the greater accumulated stress on plantar soft tissues caused by high volume of exercise, especially weight-bearing exercise, may increase the degree of compression of plantar tissue, and the occlusion duration of microvessels. The impaired plantar microcirculation under the accumulated stimulation of repeated mechanical loading may be more prone to cause tissue damage to the fragile foot tissue in people with type 2 diabetes (Chao et al., 2011). Particularly in people with diabetes and peripheral neuropathy, the dysfunction in the regulation of microvascular system, dry skin and musculoskeletal deformity caused by peripheral neuropathy can increase the vulnerability of plantar tissue to compressed damage during these physical activities (Mueller and Maluf, 2002; Jan et al., 2013b; Pu et al., 2018). Therefore, exploring the long-term effects of weight-bearing exercise with high exercise volume on the plantar microcirculation and soft tissue hardness in the diabetic foot may help to understand the risk of developing DFUs. This information may be used to develop appropriate exercise plans for people with type 2 diabetes.

The aim of this study was to compare the difference of plantar microcirculation and tissue hardness in people with type 2 diabetes who performed long-term weight-bearing exercise at high and low exercise volume. We hypothesized that participants with type 2 diabetes in the high exercise volume group would have better oxygen supply to the plantar foot and lower plantar hardness compared to the low exercise volume group.

## Materials and Methods

This is a cross-sectional observation study designed to explore the difference of plantar microcirculation and tissue hardness (the important factors in the development of DFUs) of the foot of people with diabetes who had habitual physical activity at high and low levels of exercise volume.

This study was conducted in accordance with clinical protocols approved by the institutional review board of Affiliated Hospital of National Research Center for Rehabilitation Technical Aids (20190101) and the Declaration of Helsinki (2013 revision). All participants were briefed on the study purposes and procedures and gave written informed consent prior to participation.

### Participants

A total of 130 people with diabetes confirmed their willingness to participate in this study through a public recruitment drive in the local communities and hospitals. The inclusion criteria were: 1) diagnosed type 2 diabetes, 2) ≥40 ages, 3) no symptoms such as redness, callus, inflammation, or wounds on the skin of the feet or legs, and no history of amputation, 4) no diseases such as systematic inflammation, lower extremity edema, malignant tumor, and 5) performed regular physical activities over the course of 1 year with at least 150 min/week, with no more than two consecutive days without activity (Association, 2020) before being enrolled in this study. A total of 104 participants met the inclusion criteria and were enrolled in this study.

### Physiological Information Recording and Assessment

Demographics and medical history were discussed and recorded at the initial assessment. In this study, 10 g Semmes-Weinstein monofilament and vibration perception threshold testing were used to evaluate whether participants had sensory neuropathy. For this test, 10 g monofilament was compressed perpendicular to the four areas of foot (1^st^, 3^rd^, and 5^th^ metatarsal heads and distal hallux) for 1 s and then removed. It was considered normal large-fiber nerve function if the patient could feel the touch of the monofilament at all four areas. Moreover, a biothesiometer was placed over the dorsal hallux and the amplitude of vibration was increased until participants could detect it. The protective sensation was considered normal if a participant’s vibration perception threshold was smaller than 25 V (Boulton et al., 2008). No abnormal test would rule out diabetic peripheral neuropathy. Otherwise, a participant was confirmed as a diabetic with peripheral neuropathy (Boulton et al., 2008; Schaper et al., 2020). Care was taken to avoid performing the test on callous tissue.

### Assessment of Physical Activity

The type, frequency and duration of weekly physical activity performed over the course of 1 year was recorded for each participant. This was assessed using the International Physical Activity Questionnaire (IPAQ) that has been proven to be a validated tool for physical activity assessment (Mynarski et al., 2012). The level of metabolic equivalent (MET) rating was determined based on the compendium of physical activities (Ainsworth et al., 2011), including step counts, duration and distance travelled, and the type of exercise described by the participants (Mynarski et al., 2012; Lalli et al., 2013; Ainsworth, 2014). All recorded activities and corresponding MET values in this study were as follows: walking (2.5 mph-2.8 mph, and 3 Mets), brisk walking (3.5–4.0 mph, 4.3 Mets), square dancing (5 Mets), table tennis (4 Mets), tennis (4.5 Mets), golf (4.8 Mets), billiards (2.5 Mets), cycling (4 Mets), and Tai Chi (3 Mets) (Ainsworth et al., 2011).

The weekly sum of each participant’s energy expenditure through physical activity was calculated using the Eq. 1 (Knowler et al., 2002). Diloreto et al. recommended that 27 Mets·h/week can be a reasonable target of energy expenditure for sedentary people with diabetes due to its great benefits associated with HbA1c, BMI, heart rate, and 10-years coronary heart disease risk (Di Loreto et al., 2005). Therefore, people with diabetes in this study were classified into the high exercise volume (HEV) group and the low exercise volume (LEV) group according to whether their energy expenditure exceeded 27 Mets·h/week.

$$\text{Energy Expenditure}=\sum {\text{Met}}_{i}\times {\text{T}}_{i}$$ (1)

In which, i represents different activity models, Met_i_ represents the metabolic equivalent rating corresponding to different activities, and T_i_ represents the time spent in different activities.

### Assessment of Plantar Microcirculation and Tissue Hardness

All tests were performed in a climate-controlled room at 24°C with participants in a supine position. Every participants started with a 30 min resting period before measurements. A Shore durometer (Model 1,600, Type OO, Rex Co., Buffalo Grove, United States) was used to measure the tissue hardness in the plantar regions (big toe, little toes, medial metatarsal, middle metatarsal, lateral metatarsal, medial arch, lateral arch, medial heel, and lateral heel) of each participant’s right foot. It was designed to test the hardness of soft materials such as animal tissue, foams, sponge rubber, and gels. The similar durometer has been used in several studies to assess plantar hardness in people with diabetes (Thomas et al., 2003; Periyasamy et al., 2012).

**FIGURE 1:**
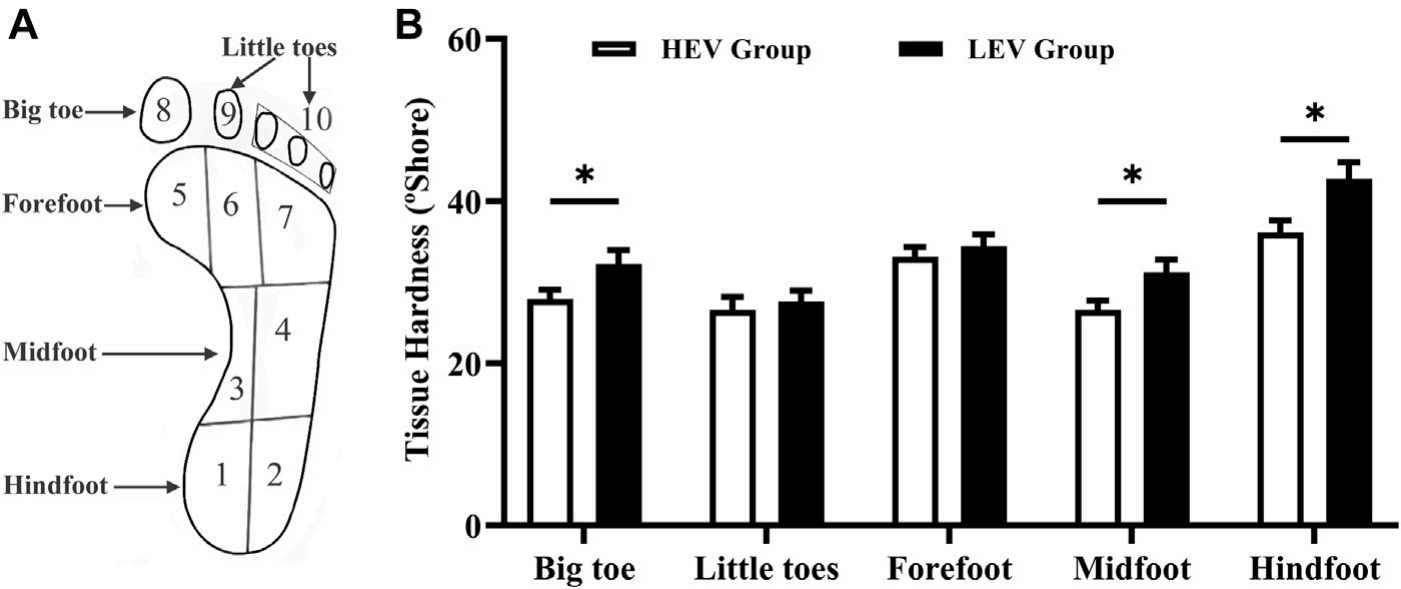
**(A)** Division of the foot. **(B)** Results of tissue hardness of plantar tissue in participants (Mean with SEM). The tissue hardness of the little toes was the average of area 8 and area 9; the tissue hardness of the forefoot was the average of area 5, area 6 and area 7; the tissue hardness of the midfoot was the average of area 3 and area 4; the tissue hardness of the hindfoot was the average of area 1 and area 2. HEV: High Exercise Volume (≥27 Mets·h/week); LEV: Low Exercise Volume (<27 Mets·h/week); SEM: standard error of mean. * indicates a significant difference between the HEV group (*n* = 45) and LEV group (*n* = 35) (*p* < 0.05).

During measurement, the durometer was pressed perpendicular to the plantar skin surface and expresses the hardness in degrees of Shore (unit: °shore). A lower Shore value indicates a softer material. Each region was measured 5 times sequentially and the mean was calculated for comparisons. Care was taken to avoid testing areas with prominent bones or callus tissue. The tissue hardness of the little toes was the average of the four little toes. The tissue hardness of the forefoot region was the average of the medial metatarsal, middle metatarsal and lateral metatarsal. The tissue hardness of the midfoot region was the average of the medial arch and lateral arch. The tissue hardness of the hindfoot region was the average of the medial heel and lateral heel (Figure 1).

After measuring the plantar tissue hardness, a moorVMS-OXY monitor (Probe OP17-1,000, Moor Instruments, Axminster, United Kingdom) was used to monitor the plantar microcirculation. This device uses a white light spectroscopy method and transmits the 6 mW white light (400–700 nm wave length) into tissue via fiber optics in order to assess tissue oxygen saturation and temperature. The probe was attached to the skin surface of the right plantar big toe area with adhesive tapes to limit movement artefacts during the measurement. The plantar tissue oxygen saturation (SO_2_) and skin temperature (Temp) of each participant in the supine position were recorded for 2 min (Newton et al., 2005; Ladurner et al., 2009).

### Sample Size

The required sample size was calculated using Power Analysis and Sample Size (PASS 15) software set for *t* test. This study assumed that the mean and standard deviation (SD) of cutaneous oxygen saturation in people with diabetes was equivalent to that of a prior study (64.1 ± 4.0%) (Kabbani et al., 2013), and the mean difference between two groups was equivalent to that of Charles et al.’s study (3.9%) (Ezema et al., 2019). A minimum of 24 participants per group was needed at a power of 90% and an alpha level of 0.05. This study assumed a drop-out rate of 20%, and considered that more than half of the participants may not regularly perform physical activities over the course of 1 year with at least 150 min/week (one of the inclusion criteria) (Wen et al., 2011). Therefore, at least 120 participants were recruited for this study.

### Data and Statistical Analyses

The mean values of SO_2_ and Temp from each participant’s right plantar big toe region, and the mean values of tissue hardness in five plantar regions (big toe, little toes, forefoot, midfoot, and hindfoot) of each participant’s right foot, were calculated. Considering that peripheral neuropathy is an important factor in contributing to the development of diabetic foot ulcers (Armstrong et al., 2017), this study also preliminarily observed differences in the mean values of SO2, Temp and plantar soft tissue hardness between people with diabetic peripheral neuropathy and people without diabetic peripheral neuropathy. In addition, plantar soft tissue may be subjected to different levels of accumulated pressure stimuli under various exercise types, which may be related to the occurrence and development of diabetic foot ulcers (Burnfield et al., 2004; Lam et al., 2019). This study conducted a preliminary comparison of the mean values of SO2, Temp and plantar soft tissue hardness among people with different exercise types.

An independent *t* test or Mann-Whitney U test (based on the normality of the variables, as tested by a Shapiro-Wilk test) was used to evaluate differences in microcirculation and plantar hardness between the HEV group and LEV group. A Spearman or Pearson correlation analysis (based on the normality of the variables, as tested by a Shapiro-Wilk test) was used to test the relationship between tissue hardness and microcirculation. The results were expressed as mean ± SD. A statistical significance level of 0.05 was used. All statistical analyses were performed in SPSS (Version 26.0, IBM, Armonk, NY, United States).

## Results

**FIGURE 2:**
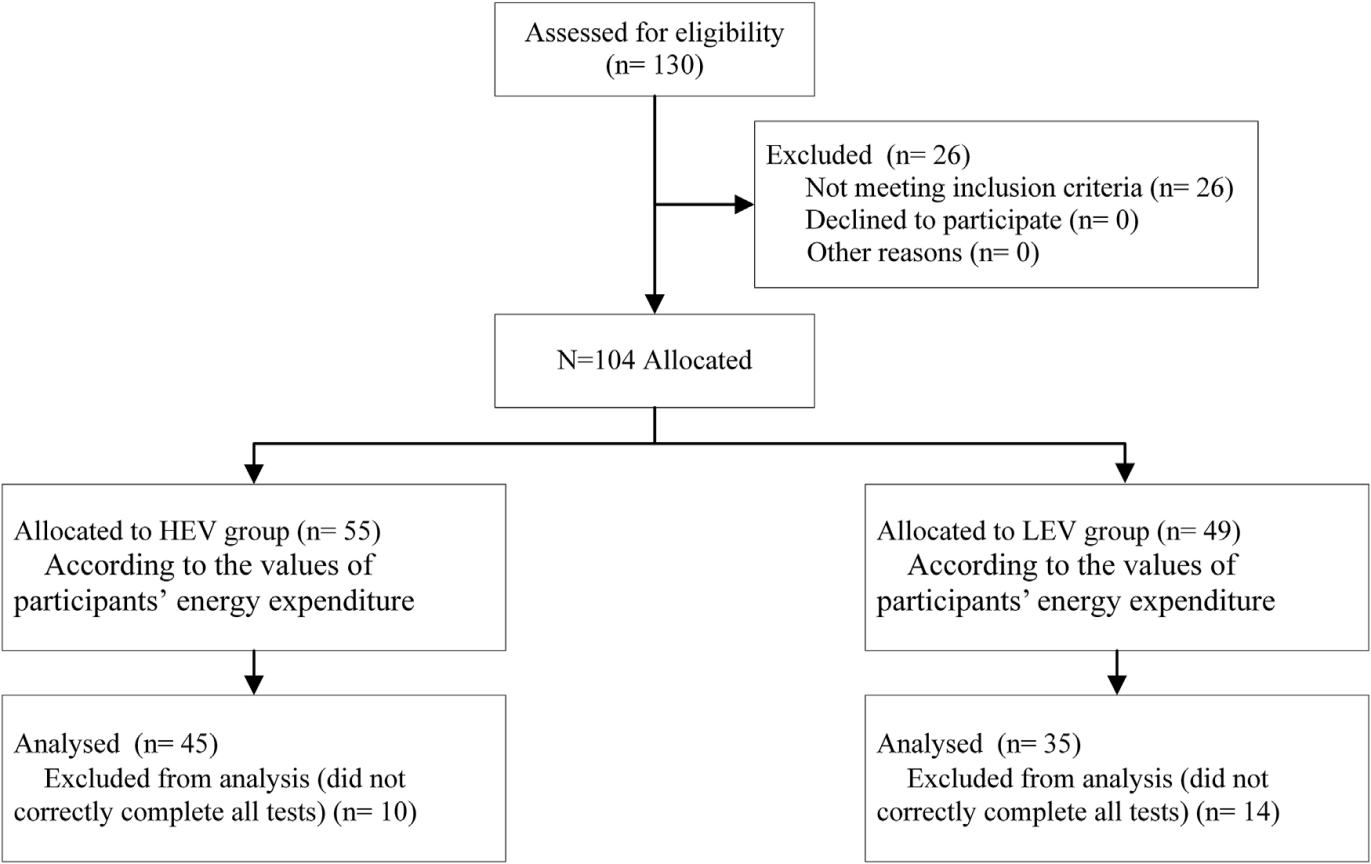
Enrollment diagram of all participants.

A total of 80 participants completed all tests (Figure 2). Among them, 45 people with diabetes were classified as the HEV group, including eight people with DPN. The remaining 35 participants were classified as the LEV group, including five people with DPN. Participant characteristics are shown in Table 1. There was no significant difference in these parameters (age, body mass index, systolic blood pressure, diastolic blood pressure, heart rate, duration of diabetes, fasting blood glucose, and ankle brachial index) between the HEV group and LEV group. The average exercise energy expenditure of the HEV group and the LEV group were 51.69 ± 21.51 Mets·h/week and 18.34 ± 4.55 Mets·h/week (*p* < 0.05), respectively (Table 1).

**TABLE 1:** Demographic and physiological information of participants in HEV and LEV groups (Mean ± SD).

Variables	HEV group	LEV group
Gender (Male/Female)	21/24	14/21
Age (years)	66.67 ± 4.55	65.85 ± 8.09
BMI (kg/m^2^)	25.57 ± 3.22	26.49 ± 3.80
SBP (mmHg)	136.17 ± 14.05	132.04 ± 14.73
DBP (mmHg)	72.51 ± 8.78	72.11 ± 9.32
Heart rate (bpm)	72.94 ± 10.91	72.54 ± 9.53
Duration of diabetes (years)	13.19 ± 9.05	11.68 ± 7.61
Fasting blood glucose (mmol/L)	7.31 ± 1.51	7.63 ± 1.60
ABI	1.08 ± 0.16	1.07 ± 0.11
Diabetic peripheral neuropathy	8 (17.8%)	5 (14.3%)

HEV: high exercise volume; LEV: low exercise volume; BMI: body mass index; SBP: systolic blood pressure; DBP: diastolic blood pressure; ABI: Ankle brachial index. There was no significant difference in all parameters between the HEV, group and LEV, group.

In the HEV group, in addition to walking, 14 of the participants without DPN routinely engaged in one or more of the following weight-bearing activities; brisk walking, square dancing, ball games (table tennis, tennis, golf, and billiards), cycling, and Tai Chi. Among them, one participant did brisk walking at about 3.7 mph every day; five participants engaged in square dancing every week; four participants engaged in ball games every week; two participants went cycling daily; and two participants did Tai Chi every day. For the other 31 participants in this group, the only routine daily physical activity was walking. Similarly, in the LEV group, the only routine physical activity was walking, but with a lower energy expenditure.

### Effects of Weight-Bearing Exercise on Plantar Microcirculation

**FIGURE 3:**
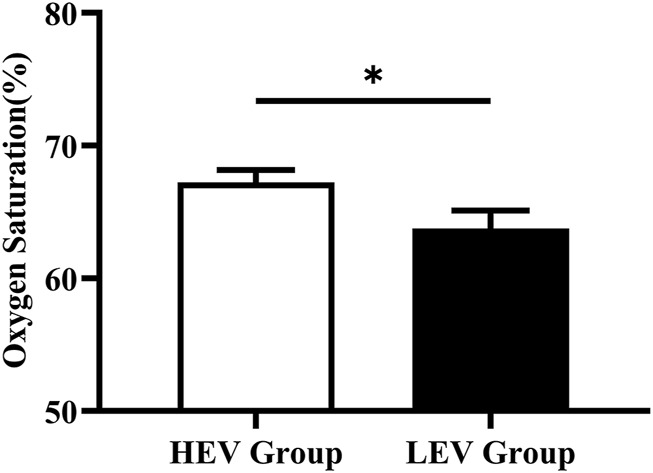
Results of oxygen saturation of plantar tissue in participants (Mean with SEM). HEV: High Exercise Volume (≥27 Mets·h/week); LEV: Low Exercise Volume (<27 Mets·h/week); SEM: standard error of mean. * indicates a significant difference between the HEV group (*n* < 45) and LEV group (*n* < 35) (*p* < 0.05).

**FIGURE 4:**
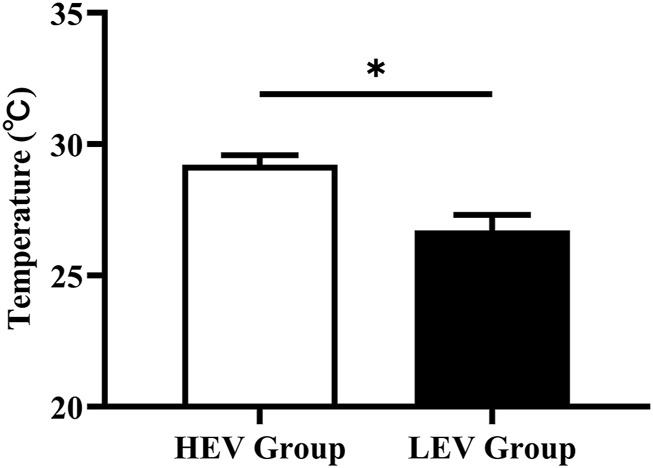
Results of skin temperature of plantar tissue in participants (Mean with SEM). HEV: High Exercise Volume (≥27 Mets·h/week); LEV: Low Exercise Volume (<27 Mets·h/week); SEM: standard error of mean. * indicates a significant difference between the HEV group (*n* < 45) and LEV group (*n* < 35) (*p* < 0.05).

The plantar oxygen saturation (SO_2_) and skin temperature (Temp) of both groups were recorded and analyzed (Figures 3, 4). The results showed that the plantar SO_2_ (67.25 ± 6.12%) and plantar Temp (29.22 ± 2.44°C) in the HEV group were both significantly higher than the LEV group (SO_2_: 63.75 ± 8.02%, *p* = 0.030; Temp: 26.72 ± 3.47°C, *p* = 0.001), respectively.

Table 2 shows the SO_2_ and Temp for participants without diabetic peripheral neuropathy (Non-DPN) and DPN. The mean SO_2_ and Temp of participants in the HEV group was higher than the LEV group. In the HEV group, the mean SO_2_ of the DPN participants was lower than the Non-DPN participants. From Table 2, it can be seen that the mean SO_2_ and mean Temp (except for Tai Chi) for participants in the sub-group “Walking + other weight-bearing activities” was higher than participants with “Only walking”.

**TABLE 2:** Plantar SO_2_ and Temp for Non-DPN and DPN participants in the HEV and LEV groups, and for Non-DPN participants performing different physical activities in the HEV group (Participants in LEV group did not engage in any form of exercise other than walking; Mean ± SD).

	HEV group		LEV group			
	Non-DPN (*n* = 37)	DPN (*n* = 8)	Non-DPN (*n* = 30)	DPN (*n* = 5)		
SO2 (%)	67.69 ± 6.37	65.23 ± 4.52	63.78 ± 8.30	63.56 ± 6.83		
Temp (°C)	29.13 ± 2.57	29.62 ± 1.76	26.77 ± 3.54	26.40 ± 3.33		

	Only walking in HEV group (n = 23)	Walking + other weight-bearing activities in HEV group				
		Brisk walking (*n* = 1)	Square dancing (*n* = 5)	Ball games (n = 4)	Cycling (*n* = 2)	Tai Chi (*n* = 2)
SO2 (%)	66.07 ± 6.33	70.90	68.28 ± 8.14	70.90 ± 5.93	73.07 ± 2.77	71.38 ± 1.81
Temp (°C)	28.87 ± 2.83	31.07	29.38 ± 2.59	29.32 ± 2.11	31.57 ± 0.03	28.02 ± 1.39

DPN: diabetic peripheral neuropathy; HEV: high exercise volume; LEV: low exercise volume; SO2: plantar oxygen saturation; Temp: skin temperature.

### Effect of Weight-Bearing Exercise on Plantar Tissue Hardness

Figure 1 compares the plantar tissue hardness between the HEV and LEV group. The results showed that the mean tissue hardness in the HEV group was lower than that of the LEV group, with a significant difference at the big toe region (HEV: 27.89 ± 7.72°Shore, LEV: 32.25 ± 9.94°Shore; *p* = 0.030), midfoot (HEV: 26.59 ± 7.59°Shore, LEV: 31.20 ± 9.30°Shore; *p* = 0.034), and hindfoot (HEV: 36.08 ± 10.17°Shore, LEV: 42.70 ± 12.23°Shore; *p* = 0.010).

It was also found that SO_2_ and Temp were negatively correlated with the tissue hardness of the big toe region (SO_2_: R = −0.299, *p* = 0.007; Temp: R = −0.311, *p* = 0.005).

Within the HEV group, the mean plantar tissue hardness of all regions of the foot for the DPN participants was higher than that of the Non-DPN participants. For DPN participants, the mean plantar tissue hardness in the HEV group was higher than the LEV group. Also, the plantar tissue hardness in the Non-DPN participants in the HEV group varied depending on the type of routine physical activities typically performed. Four participants who played ball games and one participant who did brisk walking had a higher tissue hardness in the forefoot region than other participants, while two participants who routinely cycled had the lowest tissue hardness (Table 3).

**TABLE 3:** Plantar soft tissue hardness for Non-DPN and DPN participants in the HEV and LEV groups, and for Non-DPN participants performing different physical activities in the HEV group (Participants in LEV group did not engage in any form of exercise other than walking; Mean ± SD).

		HEV group		LEV group		
		Non-DPN (*n* = 37)	DPN (*n* = 8)	Non-DPN (*n* = 30)	DPN (*n* = 5)		
Soft tissue (°shore)	Big toe	26.01 ± 6.47	36.60 ± 7.34	32.19 ± 10.43	32.60 ± 7.15		
	Little toes	24.62 ± 8.78	35.68 ± 13.90	27.55 ± 8.36	27.76 ± 7.56		
	Forefoot	31.41 ± 7.65	40.83 ± 6.48	34.45 ± 8.85	34.37 ± 7.93		
	Midfoot	24.84 ± 6.39	34.69 ± 7.80	31.53 ± 9.71	29.20 ± 6.73		
	Hindfoot	33.90 ± 9.19	46.15 ± 8.66	42.81 ± 12.84	42.04 ± 8.77		

		Only walking in HEV group (*n* = 23)	Walking + other weight-bearing activities in HEV group				
			Brisk walking (*n* = 1)	Square dancing (*n* = 5)	Ball games (*n* = 4)	Cycling (*n* = 2)	Tai Chi (*n* = 2)
Soft tissue(°shore)	Big toe	25.29 ± 6.94	29.40	28.64 ± 6.54	28.40 ± 5.93	20.10 ± 4.38	27.10 ± 1.27
	Little toes	23.94 ± 8.83	35.60	29.16 ± 8.54	27.15 ± 9.64	15.50 ± 0.99	19.60 ± 3.11
	Forefoot	30.81 ± 8.09	34.47	31.84 ± 6.19	36.03 ± 8.01	25.33 ± 4.81	32.57 ± 10.98
	Midfoot	24.76 ± 6.92	27.30	21.80 ± 4.08	28.48 ± 6.54	21.80 ± 5.23	27.85 ± 7.14
	Hindfoot	34.39 ± 10.24	28.60	30.64 ± 6.30	38.75 ± 7.24	28.90 ± 10.32	34.30 ± 8.91

DPN: diabetic peripheral neuropathy; HEV: high exercise volume; LEV: low exercise volume.

## Discussion

This study analyzed the association between exercise volume and plantar microcirculation and soft tissue hardness in people with type 2 diabetes. The results showed that participants with higher volume of habitual exercise had better oxygen supply and basal skin temperature and lower soft tissue hardness of the foot, compared to participants with lower volume of habitual exercise. These findings suggest that weight-bearing exercise with high exercise volume might be associated with better microcirculation function and softer plantar tissue, which may not increase the risk of developing foot ulcers.

Previous studies reported that exercise can increase insulin sensitivity in people with diabetes and promote the production of endothelium-dependent vasodilator nitric oxide, thus improving endothelial and microvascular function and promoting metabolism in the lower extremities (Kluding et al., 2017). Studies have also shown that a high stress stimulus can increase vessel diameter and arterial compliance, and was beneficial for the cardiopulmonary and vascular function (Huonker et al., 1996; Mueller and Maluf, 2002). Charles et al. also found that people with diabetes who engaged in an eight-week aerobics programme (bicycle) had a 3.9% increase in peripheral oxygen saturation compared to the control group (Ezema et al., 2019). Demachi et al. reported that skin temperature gradually increases with the duration of exercise (Demachi et al., 2013). Similarly, this current study found that the high exercise volume group (≥27 Mets·h/week) had higher plantar SO2 and basal Temp in comparison with the low exercise volume group (<27 Mets·h/week) (Figures 3, 4). Moreover, participants in the sub-group “Walking + other weight-bearing activities” had a higher mean SO_2_ and Temp than participants with “Only walking”. This may be due to the higher energy requirements when performing more than one routine exercise (Walking + other weight-bearing activities: 67.66 ± 29.55 Mets·h/week; Only walking: 41.54 ± 8.06 Mets·h/week). Sivanandam et al. demonstrated that the foot temperature of people with diabetes was significantly lower than that of healthy people (Sivanandam et al., 2012). Decreased skin temperature may be related to poor microvascular perfusion of the lower extremity in people with diabetes, which further aggravates microvascular dysfunction and leads to the occurrence of DFUs. The results of this study suggest that weight-bearing exercise with high exercise volume has a more positive effect on circulation and the nutrient supply to the plantar microvasculature, implying that the active weight-bearing exercise may be associated with lower risk of DFUs.

Some studies reported that the abnormal increase of plantar skin temperature may indicate the occurrence of some pathologic factors (e.g., peripheral neuropathy (Yavuz et al., 2019) and inflammation responses (van Netten et al., 2014)). However, the study of Kokate et al. demonstrated that the damage of deep tissue would occur under the pressure stimulus at temperature above 35°C in a reliable porcine model, and no damage was observed in the superficial or deep tissues with a temperature of 25°C under the pressure stimulus (Kokate et al., 1995). In this study, we compared the basal foot temperature of people with diabetes with different exercise volumes, none of the participants had a diabetic foot ulcer history and their plantar temperature did not exceed 35°C. Therefore, the mean temperature of the high exercise volume group was higher than that of the low exercise volume group, which may be due to the improvement of lower extremity microvascular perfusion in people with diabetes caused by higher volume of habitual exercise.

In this study, a OO Shore durometer was used to assess the plantar soft tissue hardness in people with diabetes. The results showed that compared to the low exercise volume group, people with diabetes in the high exercise volume group who actively engaged in weight-bearing exercise had a significantly lower plantar tissue hardness (Figure 1). According to the Physical Stress Theory proposed by Muller et al. (Mueller and Maluf, 2002; Kluding et al., 2017), tissues have different adaptive responses to external physical stress stimulation, including decreased tolerance (e.g., atrophy), maintenance, increased tolerance (e.g., hypertrophy), injury, and death. Maintenance seems to be a tissue homeostasis, physical stress stimulus below the maintenance range may result in tissue atrophy, and physical stress stimulus above the maintenance range may result in increased tolerance (Mueller and Maluf, 2002; Kluding et al., 2017). Therefore, people with diabetes in the high exercise volume group who showed a lower plantar tissue hardness may be due to the enhanced tissue adaptability under suitable repeated stress stimulus among the maintenance range. Decreased tolerance (e.g., atrophy) in the low exercise volume group may be more prone to tissue damage and cuticle thickening under mild external stress stimulus, which further leads to callus formation and an increased risk of DFUs. Some studies also reported that higher and long-term repetitive physical stress can increase collagen content and the diameter of collagen fibers, thicken skin and increase skin strength, which is beneficial to distribute plantar pressure and decrease the risk of skin breakdown (Sanders et al., 1995; Mueller and Maluf, 2002). Therefore, the mechanical stress stimulation during weight-bearing exercise in the high exercise volume group examined in this study could be expected to increase the stress tolerance threshold of plantar tissue and improve skin health.

The lower plantar tissue hardness of people with diabetes in the high exercise volume group could also be due to the improved blood circulation in the foot and enhanced protective response of tissue microvessels under stress. Mithraratne et al. demonstrated a negative correlation between the hardness of plantar tissue and the level of blood supply in the arteries of the foot (Mithraratne et al., 2012). Exercise can improve blood flow and oxygen saturation levels, which has been shown to reduce local hypoxia and waste accumulation in the foot tissue of people with diabetes (Kluding et al., 2017; Reis et al., 2019), and improve the plantar tissue viability and tolerance under an external stimulus (Jan et al., 2013b). From this study, it is evident that tissue hardness of the foot is negatively correlated with SO2 and Temp, which further confirms the above interpretation. It indicates that weight-bearing exercise with habitual high exercise volume can improve the biomechanical properties and microcirculation in the feet of people with diabetes, which can interact to play a positive role in protecting overall foot health.

In this study, eight participants were confirmed as diabetic peripheral neuropathy in the high exercise volume group (17.8%), and five participants were confirmed as diabetic peripheral neuropathy in the low exercise volume group (14.3%). This study found that the plantar SO_2_ of DPN participants was slightly lower than the Non-DPN participants in the high exercise volume group (65.23 ± 4.52 vs 67.69 ± 6.37; unit: %), and the DPN participants had the highest plantar tissue hardness value (Big toe: 36.60 ± 7.34; Little toes: 35.68 ± 13.90; Forefoot: 40.83 ± 6.48; Midfoot: 34.69 ± 7.80; Hindfoot: 46.15 ± 8.66; unit: °shore). This may be due to the reduced tissue deformability and perception to external mechanical stress caused by diabetic neuropathy. For people with diabetes and neuropathy, the loss of protective sensations in the foot, dysfunction in sweat glands, bone deformities and abnormal stress distribution would further accelerate plantar tissue stiffening (Bowering, 2001; Sun et al., 2011). The impaired microvascular regulation caused by neuropathy can hinder the oxygenation capacity and waste removal capability of foot tissue under mechanical stress. This may be the reason for the decreased SO_2_ and increased tissue hardness in DPN participants in the high exercise volume group (Edmonds et al., 1982; Stevens et al., 1991). Although it has been reported that moderate walking speed does not increase the incidence and recurrence of foot ulcers in people with DPN (LeMaster et al., 2008), a higher tissue hardness and lower SO_2_ are thought to increase the risk of developing foot ulcers (Murray et al., 1996; Jan et al., 2013a). Therefore, neuropathy may be an important consideration when people with diabetes engage in exercise, and it is necessary for people with DPN to carefully choose the type and intensity of weight-bearing exercise. In the future, it is still necessary to expand the sample size and conduct a similar study on people with diabetic peripheral neuropathy to clarify the impact of weight-bearing exercise on the risk of diabetic foot ulcers in people with diabetic peripheral neuropathy.

In addition, the results of this study showed that participants who regularly went for brisk walk and participated in ball games had higher plantar tissue hardness in the forefoot region. Participants with brisk walking had the highest plantar tissue hardness in the hindfoot region (Table 3). Burnfield et al. found that faster walking increased the plantar pressure around the toes, medial metatarsal heads and heel when healthy elderly people walked at different speeds (57, 80, 97 m/min) (Burnfield et al., 2004). Similarly, Lam et al. reported that playing table tennis produced higher peak pressure in the total foot during side-step and cross-step footwork in comparison with one-step footwork (Lam et al., 2019). This suggests that the high hardness in participants of this study who routinely participated in brisk walking and ball games (table tennis, tennis, golf, and billiards) may be related to the repetitive high plantar pressure acting on the plantar soft tissues during exercise. Such a high-magnitude pressure stimulus for a brief duration may cause excessive physical stress to plantar tissue, and further cause callus formation and tissue damage (Murray et al., 1996; Mueller and Maluf, 2002). However, the effect of different types of exercise on the risk of developing DFUs needs further exploration.

There are some limitations to this study that should be noted. Firstly, the durometer works on the principle of indentation to characterize the plantar tissue hardness, ignoring the non-linear viscoelastic behaviour and tissue thickness. Subsequent studies can use ultrasound imaging to explore the biomechanical properties of plantar tissue in more detail. Secondly, this study only considered a limited number of physical activities with moderate intensity, and the predominant exercise performed across all participants was walking. The influence of accumulated stress and different activity patterns on the risk of developing DFUs needs further study. Thirdly, the impact of exercise on other DFUs risk factors such as transcutaneous oxygen tension (TcPO_2_), the microvascular response to mechanical stress, musculoskeletal deformities and callus formation of the foot may be considered in future studies. Fourthly, participants in this study had not yet developed foot ulcers. Because the relationships between foot ulcers and tissue hardness, and oxygen saturation are still unclear, follow-up studies should determine whether lower oxygen saturation and higher plantar tissue hardness in the low exercise volume group with diabetic peripheral neuropathy is associated with higher incidence of diabetic foot ulcers.

## Conclusion

In conclusion, this study found that higher volumes of habitual weight-bearing exercise in people with type 2 diabetes are associated with better plantar tissue oxygenation and lower plantar tissue hardness. These changes may decrease the risk of developing diabetic foot ulcers.

## Data Availability

The original contributions presented in the study are included in the article/Supplementary Material, further inquiries can be directed to the corresponding authors.
